# *FAAH* and *CNR1* Polymorphisms in the Endocannabinoid System and Alcohol-Related Sleep Quality

**DOI:** 10.3389/fpsyt.2021.712178

**Published:** 2021-09-09

**Authors:** Soundarya Soundararajan, Narjis Kazmi, Alyssa T. Brooks, Michael Krumlauf, Melanie L. Schwandt, David T. George, Colin A. Hodgkinson, Gwenyth R. Wallen, Vijay A. Ramchandani

**Affiliations:** ^1^Human Psychopharmacology Laboratory, National Institute on Alcohol Abuse and Alcoholism, National Institutes of Health, Bethesda, MD, United States; ^2^National Institutes of Health Clinical Center, Bethesda, MD, United States; ^3^Office of the Clinical Director, National Institute on Alcohol Abuse and Alcoholism, National Institutes of Health, Bethesda, MD, United States; ^4^Laboratory of Neurogenetics, National Institute on Alcohol Abuse and Alcoholism, National Institutes of Health, Bethesda, MD, United States

**Keywords:** endocannabinoids, sleep phenotypes, alcohol use disorder, Pittsburgh Sleep Quality Index, actigraphy

## Abstract

Sleep disturbances are common among individuals with alcohol use disorder (AUD) and may not resolve completely with short-term abstinence from alcohol, potentially contributing to relapse to drinking. The endocannabinoid system (ECS) is associated with both sleep and alcohol consumption, and genetic variation in the ECS may underlie sleep-related phenotypes among individuals with AUD. In this study, we explored the influence of genetic variants in the ECS (Cannabinoid receptor 1/*CNR1*: rs806368, rs1049353, rs6454674, rs2180619, and Fatty Acid Amide Hydrolase/*FAAH* rs324420) on sleep quality in individuals with AUD (*N* = 497) and controls without AUD (*N* = 389). We assessed subjective sleep quality (from the Pittsburgh Sleep Quality Index/PSQI) for both groups at baseline and objective sleep efficiency and duration (using actigraphy) in a subset of individuals with AUD at baseline and after 4 weeks of inpatient treatment. We observed a dose-dependent relationship between alcohol consumption and sleep quality in both AUD and control groups. Sleep disturbance, a subscale measure in PSQI, differed significantly among *CNR1* rs6454674 genotypes in both AUD (*p* = 0.015) and controls (*p* = 0.016). Only among controls, neuroticism personality scores mediated the relationship between genotype and sleep disturbance. Objective sleep measures (sleep efficiency, wake bouts and wake after sleep onset), differed significantly by *CNR1* rs806368 genotype, both at baseline (*p* = 0.023, 0.029, 0.015, respectively) and at follow-up (*p* = 0.004, *p* = 0.006, *p* = 0.007, respectively), and by *FAAH* genotype for actigraphy recorded sleep duration at follow-up (*p* = 0.018). These relationships suggest a significant role of the ECS in alcohol-related sleep phenotypes.

## Introduction

Sleep disturbances are prevalent among individuals with alcohol use disorder (AUD). The relationship between sleep disturbances and alcohol consumption is bi-directional. Sleep disturbances contribute to the development and maintenance of AUD. Both acute and chronic alcohol consumption is associated with sleep disturbances, which persist through abstinence. The prevalence of insomnia in individuals with AUD during drinking and protracted abstinence ranges widely between 36 and 91% (1). In a recent review, Koob and Colrain (2) reported that sleep disturbances are ubiquitous in all three stages of AUD, binge/intoxication, withdrawal/negative affect, and preoccupation/anticipation. Our previous study demonstrated a high prevalence of sleep disturbances among participants with AUD on admission to a 4-week inpatient detoxification treatment program with 90.4% reporting sleep disturbance within 2 days of admission, as well as a significant average reduction in the severity of sleep disturbance. Yet, some residual sleep disturbances persisted after 4 weeks of inpatient treatment, with 50.5% reporting sleep disturbance by day 28 (3). Sleep disturbances may be a risk factor for relapse to drinking (4), and therefore, identifying the factors that put individuals most at risk for persistent sleep disturbances and potential relapse is an interesting and clinically important goal.

Studies demonstrate that factors such as age (middle aged, older men and women), race/ethnicity, (lower) socioeconomic status, male sex, and obesity contribute to sleep disturbances in the general population (5–8). Additionally, recent genome-wide association studies (GWAS) provide evidence of an association between sleep disturbance and genetic factors (9, 10). With underlying genetic vulnerability to sleep disturbances, quantity, and frequency of alcohol consumption and associated comorbidities may further exacerbate the risk for poor sleep quality.

Several neurotransmitter systems are implicated in the neurobiology of alcohol and sleep, and of these, the endocannabinoid system (ECS) is of particular interest. The potential role of ECS in addictive disorders is not restricted to cannabis use disorder. A recent study suggested that genetic variants in the ECS were associated with the development of AUD in adolescents (11). ECS contributes to alcohol reinforcement and consumption, and chronic consumption alters the endocannabinoid system (12). A key enzyme in the metabolism of the endocannabinoid anandamide is Fatty Acid Amide Hydrolase or FAAH, encoded by the *FAAH* gene. Knockout models of *FAAH* and pharmacological inhibition led to increased preference for alcohol and increased alcohol consumption in preclinical studies (13). In humans, a single nucleotide polymorphism (SNP) in the *FAAH* gene rs324420 (c.385C>A or Pro129Thr) substitutes proline to threonine, making the FAAH protein vulnerable to degradation. This reduces steady-state FAAH levels leading to increased endocannabinoid activity. This functional c.385C>A SNP has also been associated with increased alcohol consumption, which may increase the risk of AUD (14).

Endocannabinoids act via Cannabinoid Receptor Type-1 (CB1R) and Type-2 (CB2R) that are expressed primarily in the brain and periphery, respectively (15). CB1R, the most abundant metabotropic receptor in the brain, is highly expressed in regions related to motivation and cognition (prefrontal cortex, amygdala, hypothalamus) and reward processing (nucleus accumbens, ventral tegmental area, ventral pallidum, and caudate putamen) (16). The G-protein coupled CB1 receptor is encoded by *CNR1* gene localized at chromosome 6q14-q15 (17). Animal models in which CB1 is knocked out exhibited reduced voluntary alcohol consumption and self-administration (18, 19). Alcohol administration increases endocannabinoid levels extracellularly in the nucleus accumbens (20). Chronic alcohol consumption compromises endocannabinoid signaling by decreasing CB1R expression in the brain and reducing its affinity for G-protein (21). Studies examining the role of the endocannabinoid system in sleep have found that CB1 agonists promote sleep and administration of CB1 antagonists increases time spent in active wakefulness in animal models (22, 23).

With demonstrable evidence for the potential role of the endocannabinoid system influencing AUD and sleep, it is vital to identify genetic risk factors related to this triad. This could deepen our understanding of the genetic underpinnings underlying sleep disturbances in AUD and may have implications for treatment. In this context, Maple et al. examined genetic variants in the *FAAH* and *CNR1* genes in sleep phenotypes among cannabis users; and observed that the *FAAH* C/C genotype rs324420 was associated with poor sleep and that depressive symptoms mediated the relationship between sleep and FAAH genotype (24). Also, *CNR1* variations have been explored in association with personality domains, and there have been reports of significant single gene, epistatic effects, and haplotype associations with neuroticism and its facets (25). To our knowledge, no studies have directly examined the relationship between the endocannabinoid system and sleep in AUD.

Thus, the goal of this study was to examine the role of genetic variation in the endocannabinoid system on sleep-related measures in individuals with AUD and non-affected controls. Specifically, we examined the following research questions: (1) Is alcohol associated with sleep quality? (2) Are genetic variants in *FAAH* and *CNR1* genes associated with subjective and objective sleep quality? (3) If there are genetic differences, are they mediated by anxiety and depressive symptoms or related personality traits?

## Materials and Methods

### Description of Study Population

We selected individuals undergoing inpatient treatment for AUD enrolled in screening and assessment clinical trials (NCT#00106093 & NCT#02231840), and also non-treatment-seeking individuals, including participants with and without AUD, enrolled in two different screening protocols (NCT#02231840 & NCT#00001673). The protocols were approved by the NIH Addictions Institutional Review Board. DSMIV or DSM5 AUD diagnoses were based on the Structured Clinical Interview for The Diagnostic and Statistical Manual Of Mental Disorders (SCID-IV or SCID-5). Participants were excluded from this analysis if SCID data were not available or they only had a SCID diagnosis of the past (but not current) AUD. A measure of sleep quality (PSQI) was administered on day 2 for treatment-seeking individuals with AUD and on the screening day for non-treatment seeking and healthy controls. A written informed consent was obtained from all the participants prior to the study. Participants flowchart is depicted in Supplementary Figure 1.

### Description of Measures

#### Structured Clinical Interview for the Diagnostic and Statistical Manual of Mental Disorders (SCID)

The SCID consists of 11 modules evaluating criteria for psychiatric diagnoses. Trained professionals conducted SCID interviews, and final DSM-IV/DSM-5 (Diagnostic and Statistical Manual of Mental Disorders, version IV OR version 5) diagnoses were determined through a consensus process involving psychiatrists (26, 27). SCID diagnoses include mood and anxiety disorders, substance use disorders, and other psychiatric disorders that are frequently co-morbid with AUD.

#### Timeline Followback (TLFB) Measures for Alcohol Consumption

The TLFB assesses alcohol drinking patterns and amounts over a fixed interval, usually 90 days (28). The assessment collects drinking information using personal historical events as clues and the number of items corresponding to the number of days of interest (29). The TLFB was typically administered during the first week of inpatient stay for the treatment group and on the screening day for the non-treatment group. Drinking data were collected for the 90 days preceding admission/screening. The primary outcomes of interest from the TLFB are total drinks, average drinks per day, number of drinking days, and the number of heavy drinking days.

#### Comprehensive Psychopathological Rating Scale (CPRS)

The CPRS is a 19-item measure that assesses the severity of psychiatric symptoms and observed behaviors (30–32). Two subscales from the CPRS include the Brief Scale for Anxiety (BSA) and Montgomery-Asberg Depression Rating Scale (MADRS). The BSA measures pathological anxiety with or without other medical or psychological disorders, and the MADRS assesses symptoms of depression. The BSA and MADRS were administered weekly during the inpatient stay to the treatment group.

#### Alcohol Use Disorders Identification Test (AUDIT)

The AUDIT, is a ten-item questionnaire developed by the World Health Organization (WHO) to identify harmful alcohol drinking pattern (33). It screens for excessive drinking in three domains: alcohol intake, potential dependence and the experience of alcohol-related harm. AUDIT is relevant for brief interventions to avoid consequences of alcohol drinking. The scores range from 0 to 40, higher scores indicate greater alcohol severity. AUDIT scores were available for 77% of our study sample (*n* = 681, 381 controls, and 300 AUD cases) as the AUDIT was not assessed in a subset of the study population.

#### Alcohol Dependence Scale (ADS)

This 25-item scale quantitatively assesses the severity of alcohol dependence in a variety of clinical settings. The questionnaire takes about 5–10 min to complete and was administered during the first week of inpatient stay for the treatment group and on the screening day for the non-treatment group. The scale covers alcohol withdrawal symptoms, impaired control over drinking, awareness of a compulsion to drink, increased tolerance to alcohol, and salience of drink-seeking behavior. A score of 9 or more is highly predictive of DSM diagnosis of alcohol dependence (34).

#### Smoking History Questionnaire (SMHQ)

The Smoking History Questionnaire (SHQ) is a 13-item questionnaire that assesses various aspects of smoking behavior such as daily smoking pattern, number of years smoking, other forms of nicotine use, smoking history of family, and age of first cigarette. The questionnaire takes about 4–5 min to complete and was administered during the first week of inpatient stay for the treatment group and on the screening day for the non-treatment group. One of the variables from SHQ used in this analysis is pack-years, which is calculated by multiplying the number of packs of cigarettes smoked per day by the number of years the person has smoked (35).

#### NEO Personality Inventory (NEO PI-R)

The Neuroticism-Extroversion/Introversion-Openness to Experience (Five-Factor) Personality Inventory-Revised (NEO PI-R) is a 240-item self-administered measure that provides scores on various dimensions of personality (36, 37). The NEO-PI-R was administered around day 21 of the inpatient stay for the treatment group and on the screening day for the non-treatment group. The assessment yields scores in five domains; Neuroticism (N), Extraversion (E), Openness to Experience (O), Agreeableness (A), and Conscientiousness (C). Each domain includes six facets, higher scores indicate higher facet measured. Neuroticism domain includes traits including anxiety, angry-hostility, depression, self-consciousness, impulsiveness, and vulnerability.

#### The Pittsburgh Sleep Quality Index (PSQI)

The PSQI is a validated self-administered questionnaire that measures sleep quality and disturbance over one month (38–40). Nineteen items generate a global score with seven component subscales, each subscale being scored from 0 to 3 with 0 representing no sleep disturbance. A global PSQI score 5 or greater indicates poor sleep quality. The PSQI was completed on day 2 of the inpatient stay in the treatment group and on the screening day in the non-treatment group. The treatment group completed another PSQI assessment after 4 weeks of inpatient treatment.

#### Actigraphy

Actiwatches® are small actigraphy-based devices that record digitally-integrated measures of gross motor activity. For a subset of the treatment group, the *Philips Actiwatch-2* model (Philips Respironics, Bend, Oregon) collected objective data on sleep quality and duration, and daytime activity patterns. Compared to polysomnography, actigraphy-recorded sleep variables demonstrate high sensitivity and moderate accuracy for detecting sleep in populations with normal and disturbed sleep (41, 42). Participants wore the Actiwatch on their non-dominant wrist from the day after admission until discharge from the inpatient unit. Actiwatch data were downloaded and analyzed using the *Actiware*® software (Respironics Actiware v.6.0.9; Philips Respironics, Bend, Oregon). Daily sleep diaries were used to clean and adjust sleep data if necessary. The following outcomes were used in this analysis: weekly averages of sleep efficiency, wake after sleep onset (WASO), sleep duration, time in bed, and sleep onset latency (3).

#### SNP Selection and Genotyping

*CNR1* and FAAH genes were selected from the ECS pathway based on their associations with sleep and alcohol-related phenotypes either in human or animal model-based genetic studies (18, 19, 24, 43–52). We restricted our SNP selection to functional variants and those that show relevance to alcohol or substance-related phenotypes identified from previous genetic association studies. The following SNPs were genotyped on arrays: *FAAH* rs324420 (14, 53, 54), *CNR1* rs806368 (54–56), *CNR1* rs1049353 (53, 57–60), *CNR1* rs6454674 (59, 60), and *CNR1* rs2180619 (24, 61, 62). Genotyping was performed on whole blood extracted DNA by Illumina OmniExpress BeadChip array (Illumina San Diego, California, USA) at the Laboratory of Neurogenetics, NIAAA. Ancestry was measured via a large panel of Ancestry Informative Markers (AIM scores) (63). For all analyses, we used the dominant model because of the modest numbers in the minor homozygotes group. Thus, for the *FAAH* rs324420, AA homozygotes and AC groups were combined to A carriers and compared with CC homozygotes; *CNR1* rs806368: G carriers vs. AA homozygotes, *CNR1* rs1049353: A carriers vs. AA homozygotes; *CNR1* rs6454674: C carriers vs. AA homozygotes; and *CNR1* rs2180619: A carriers vs. GG homozygotes.

### Statistical Analysis

Initial analyses, using independent *t*-tests, were conducted to examine whether the baseline demographic variables and the sleep quality differed between individuals with AUD (i.e., cases, *n* = 497) and individuals without AUD (i.e., controls, *n* = 389). Paired *t*-tests examined whether the sleep quality measures changed after 4 weeks of inpatient treatment by comparing the baseline and 4-week timepoints in the treatment group subset (*n* = 252). For genotype data, Hardy-Weinberg equilibrium was evaluated in cases and controls by chi-square tests. Fisher's Exact test was used to assess if the genotypes were differentially distributed among cases and controls. All baseline demographic variables were tested for differences among genotypes for inclusion as covariates in subsequent analysis. All research questions and corresponding statistical approaches are detailed below and depicted in Supplementary Figure 2.

#### Research Question 1: Is Alcohol Associated With Sleep Quality?

To test whether alcohol consumption and frequency measures were related to sleep quality, we ran correlations and examined the direction of correlation and the correlation coefficients among drinking-related variables and global PSQI scores separately for the AUD and control groups.

#### Research Question 2: Are Genetic Variants in FAAH and CNR1 Genes Associated With Subjective and Objective Sleep Quality?

To examine if there were any interactive effects of AUD status (AUD or control) and genotype on PSQI sleep quality, we conducted a two-way ANCOVA on PSQI sleep quality measures: The dependent variable in each model was global or a subscale PSQI score. AUD status (2 levels: AUD and controls) and genotypes were treated as independent variables. AUD status and genotypes were tested for interactions. Separate models were run for all five genotypes. In each model the covariates were as follows: Age, sex, years of education, pack-years, Alcohol Dependence Severity (ADS) scores, Ancestry Informative Markers for Africa & Europe, presence of mood disorders, presence of anxiety disorders, presence of cannabis use disorder, and any other substance use disorder other than nicotine. We used Levene's test to check whether homogeneity of variance was met. As we observed interactive effects only for one of the subscales (sleep medication use) only for *CNR1* rs1049353 genotype, we ran separate ANCOVA models for the AUD and the control groups.

To test the genotype effect on PSQI sleep quality measures (global and each subscale scores), we ran a one-way ANCOVA separately in the AUD (*n* = 497) and control groups (*n* = 389). The dependent variable in each model was global or a subscale PSQI score. Genotypes were treated as independent variable. Separate models were run for all five genotypes. In each model the covariates were as follows: Age, sex, years of education, pack years, Alcohol Dependence Severity (ADS) scores, Ancestry Informative Markers for Africa & Europe, presence of mood disorders, presence of anxiety disorders, presence of cannabis use disorder, and any other substance use disorder other than nicotine. We used Levene's test to check whether homogeneity of variance was met.

Similarly, for the actigraphy-based objective sleep measures, we performed a one-way ANCOVA to examine genotype effects after controlling for the covariates mentioned above. In these models, baseline models included week one average of actigraphy collected data (sleep efficiency, WASO, sleep duration, time in bed, and sleep onset latency) as dependent variables and genotypes as independent variables. Whereas, in the models using the follow-up score, week four average actigraphy scores were treated as dependent variables and genotypes as independent variables.

#### Research Question 3: If There Are Genetic Differences, Are They Mediated by Anxiety and Depressive Symptoms or Related Personality Traits?

For testing the mediation models, we included only one genotype *CNR1* rs6454674, as we observed that the sleep disturbances subscale (of the PSQI) significantly differed between the *CNR1* rs6454674 genotypes. To identify *CNR1* rs6454674 genotype-based differences, we ran independent *t*-tests on the baseline anxiety and depression scores from the CPRS, and personality measures from the NEO-PI. We found that only among controls, the neuroticism domain of the NEO-PI and two of its subsets, vulnerability to stress and self-conscientiousness, were significantly different between the genotype groups. So, we conducted a mediation analysis only among controls, to explore whether neuroticism acts as a mediator between the genotype and sleep disturbances with Hayes's Process v3.3 in SPSS (64).

All significant values are reported at the significance level of *p* < 0.05. All analyses were performed in SPSS, and figures were generated with GraphPad Prism 7.00, for Windows GraphPad Software (La Jolla California USA, www.graphpad.com) and RStudio ggplot2 package (65, 66).

## Results

### Demographics and Clinical Characteristics

A total of 886 participants completed PSQI at baseline; 389 were classified as controls, and 497 were classified as cases (i.e., participants with AUD). Of the participants with AUD, 76% (*n* = 378) were treatment-seeking. Fifty-two percent (*n* = 252) of the individuals with AUD were reassessed for sleep quality using PSQI at the end of the inpatient stay (mean 26.09 ± 1.9 days). At baseline, the participants with AUD were relatively older and more likely to be male and had fewer years of education than controls. As expected, participants with AUD had higher AUDIT scores, pack-years of smoking, and drinking measures by TLFB (Table 1).

**Table 1:** Sociodemographic and drinking characteristics of participants at baseline.

**Baseline characteristic**	**Controls**		**AUD**		***P***
	***N***	**Mean ± SD**	***N***	**Mean ± SD**	
Age	389	36.83 ± 12.9	497	43.55 ± 11.6	<0.001
Sex (Male: Female)^a^	193:196	-	347:150	-	<0.001
Years of education	379	15.75 ± 3.6	487	13.78 ± 2.8	<0.001
AUDIT	381	3.26 ± 3.5	300^b^	23.73 ± 9.2	<0.001
TLFB total drinks	384	50.14 ± 121.4	480	876.96 ± 713.2	<0.001
TLFB drink days	384	16.91 ± 20.8	480	68.29 ± 23.6	<0.001
TLFB average drinks per day	384	1.92 ± 1.8	480	12.34 ± 7.9	<0.001
TLFB heavy drink days	384	3.26 ± 10.4	480	60.09 ± 28.9	<0.001
ADS score	375	1.23 ± 2.2	480	18.37 ± 9.0	<0.001
Pack years	381	0.74 ± 4.3	486	7.78 ± 11.7	<0.001

*Diagnosis based on The Diagnostic and Statistical Manual of Mental Disorders (4th/5th ed.).*

*AUD, Alcohol Use Disorder; AUDIT, Alcohol Use Disorders Identification Test; TLFB, Timeline Followback; ADS, Alcohol Dependence Scale.*

a *Chi-squared test.*

b *A subset of AUD cases did not complete the AUDIT as part of their assessment, resulting in a lower N for this measure*.

Participants with AUD and controls differed in all PSQI-based sleep outcome measures; the mean global score for AUD was 8.80 ± 4.3, and controls had a mean score of 2 ± 2.4. PSQI global and component mean scores at baseline and follow-up are presented in Supplementary Table 1.

### Genotypes

All SNPs were in Hardy-Weinberg equilibrium in cases and controls (*p* > 0.05). Genotype frequencies and minor allele frequencies are reported in Table 2. There were no significant differences in genotypes between participants with AUD and controls. Pack-years differed among genotypes in rs806368 and rs1049353 in the full sample and in participants with AUD when analyzed separately; all other baseline demographic measures were comparable among the genotypes.

**Table 2:** Genotype frequencies.

**Genotypes**	**Controls**	**AUD**	***p* value^***a***^**	**MAF**	
				**Controls**	**AUD**
***FAAH*** **rs324420**					
A carriers	201 (52.34)	244 (49.49)	0.415	0.30	0.29
CC	183 (47.66)	249 (50.51)			
***CNR1*** **rs806368**					
G carriers	267 (68.64)	350 (70.56)	0.556	0.16	0.19
AA	122 (31.36)	146 (29.44)			
***CNR1*** **rs1049353**					
A carriers	281 (72.61)	367 (74.44)	0.590	0.14	0.15
GG	106 (27.39)	126 (25.56)			
***CNR1*** **rs6454674**					
C carriers	168 (45.28)	197 (41.21)	0.236	0.35	0.33
AA	203 (54.72)	281 (58.79)			
***CNR1*** **rs2180619**					
A carriers	109 (28.02)	131 (26.36)	0.595	0.48	0.50
GG	280 (71.98)	366 (73.64)			

*MAF, Minor Allele Frequency; AUD, Alcohol Use Disorder; FAAH, Fatty acid amide hydrolase; CNR1, Cannabinoid Receptor 1.*

a *p values from Fisher's exact tests*.

### Is Alcohol Associated With Sleep Quality?—Alcohol Quantity and Frequency Is Associated With Subjective Sleep Quality Both in AUD and Controls

**Figure 1:**
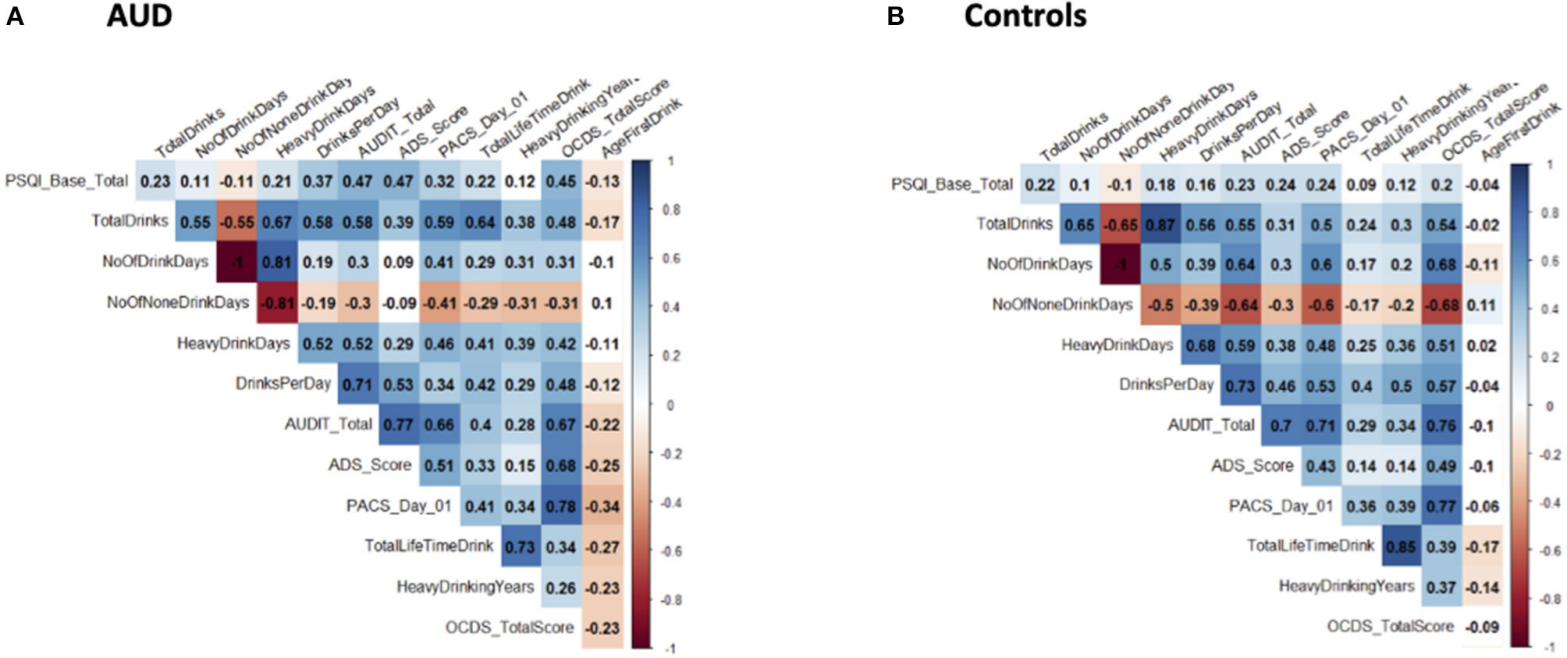
Correlograms for global PSQI score and alcohol-related measures. These correlograms depict the correlation between global PSQI scores and various alcohol-related measures in both AUD **(A)** and control **(B)** groups. Significant correlations are indicated by coloration (ranging from red for negative and blue for positive) and non-significant correlations are left uncolored. Correlation coefficients are indicated within each box.

Figure 1 shows correlograms for global PSQI scores and alcohol consumption (90-day TLFB and lifetime drinking history) and consequences (ADS, OCDS) in the AUD and control groups. We observed significant positive bivariate correlations (except for the no drinking days where the relationship was negative) amongst the global PSQI scores and alcohol measures in both AUD and control groups. The overall pattern suggested stronger correlations in the AUD group than the control group, possibly reflecting group differences in both PSQI scores and alcohol measures as reported in Table 1.

### Are Genetic Variants in FAAH and CNR1 Genes Associated With Subjective and Objective Sleep Quality?

In the two-way ANCOVA, there was a statistically significant interaction between AUD status and *CNR1* rs1049353 genotype only on self-reported sleep medication use [a subscale of PSQI), [*F*_(1,828)_ = 5.253, *p* = 0.022, partial η^2^ = 0.006]. Simple main effects were examined with statistical significance receiving Bonferroni adjustment and being accepted at *p* < 0.025 level. There was a statistically significant difference in *CNR1* rs1049353 genotypes among participants with AUD, with the GG homozygotes reporting greater medication use than A carriers (*p* = 0.008) (Supplementary Figure 3).

#### Subjective Sleep Disturbances Differed by CNR1 rs6454674 Genotype at Baseline in Both Participants With AUD and Controls

**Figure 2:**
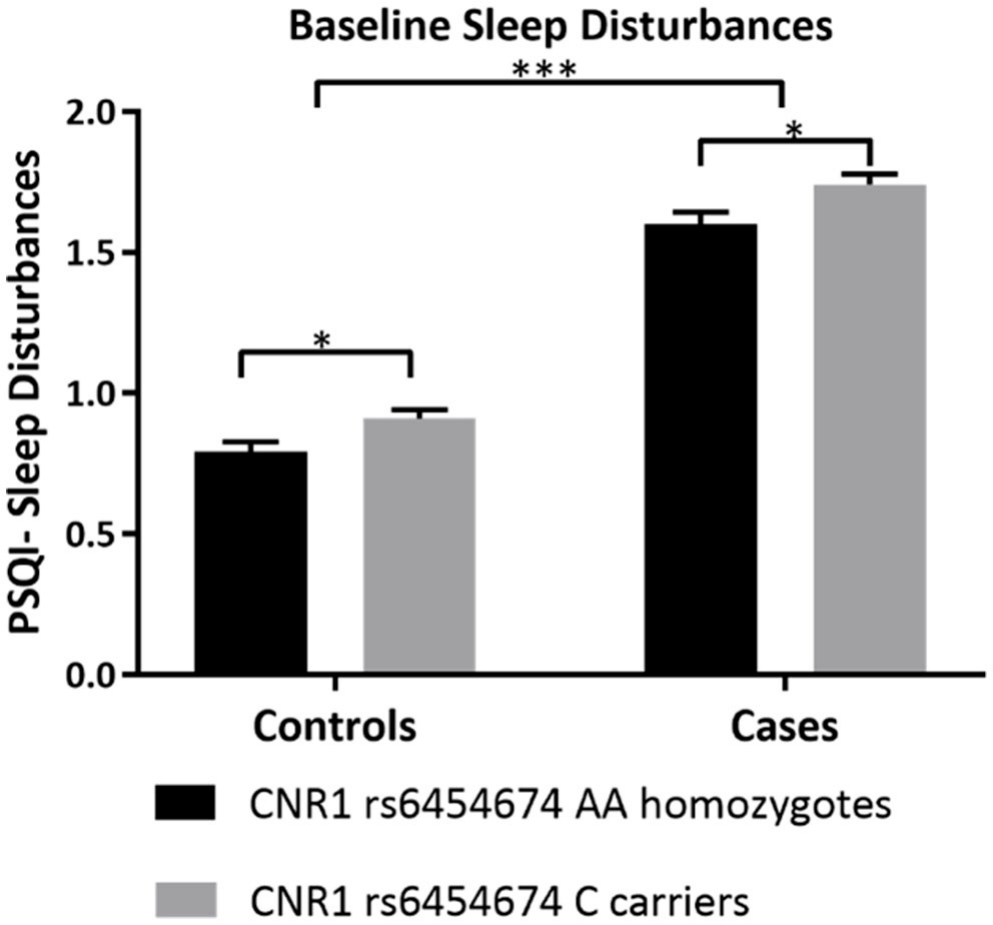
Differences in sleep disturbances by CNR1 rs6454674 Genotype at Baseline. This figure demonstrates that, at baseline, C allele carriers (CC/AC genotypes) of *CNR1* rs6454674 had greater sleep disturbances compared to AA homozygotes. The results are from the two-way ANCOVA tests run for genotype*AUD status (AUD/No AUD) after controlling for age, sex, years of education, pack years, Alcohol Dependence Severity (ADS) scores, Ancestry-informative marker (AIM scores for Africa, Europe), presence of mood disorders, and/or anxiety disorders, presence of Cannabis Use Disorder, and/or any Substance Use Disorder other than nicotine (controls: [*F*_(1,344)_ = 5.832, *p* = 0.016, partial η^2^ = 0.017], *N* = 357 and AUD: [*F*_(1,443)_ = 6.017, *p* = 0.015, partial η^2^ = 0.013, *N* = 456]. Bars represent adjusted means and error bars denote standard errors. Higher scores indicate poorer sleep quality in PSQI. PSQI, Pittsburgh Sleep Quality Index. *p <0.05, ***p <0.001.

After adjusting for covariates, one-way ANCOVA run separately in controls and participants with AUD, showed that there was a statistically significant difference in the sleep disturbances subscale of PSQI (all PSQI subscale scores range from 0 to 3) between the *CNR1* rs6454674 genotypes, controls: [*F*_(1,344)_ = 5.832, *p* = 0.016, partial η^2^ = 0.017] and participants with AUD: [*F*_(1,443)_ = 6.017, *p* = 0.015, partial η^2^ = 0.013]. The sleep disturbances subscale scores were higher in C carriers, which was significantly greater than AA homozygotes [controls: mean difference of 0.119 (95% CI, 0.022, 0.216) and participants with AUD: mean difference of 0.141 (95% CI, 0.028, 0.255)] (Figure 2). Mean scores (unadjusted and adjusted for covariates) are provided in Supplementary Table 2.

Among controls, age, ADS score and presence of mood disorders were also significantly different between *CNR1* rs6454674 genotype groups: C carriers reported greater age: [*F*_(1,344)_ = 11.922, *p* = 0.001, partial η^2^ = 0.033], greater ADS score: [*F*_(1,344)_ = 10.460 *p* = 0.001, partial η^2^ = 0.030], more mood disorders: [*F*_(1,344)_ = 4.791, *p* = 0.029, partial η^2^ = 0.014] than AA homozygotes.

Among participants with AUD, the covariates, sex, age, ADS score and presence of mood disorders were also significantly different, Among C carriers there were more males, sex: [*F*_(1,443)_ = 7.303, *p* = 0.007, partial η^2^ = 0.016], greater in age: [*F*_(1,443)_ = 13.400, *p* < 0.001, partial η^2^ = 0.029], reported greater ADS score: [*F*_(1,443)_ = 56.207, *p* < 0.001, partial η^2^ = 0.113], and more mood disorders: [*F*_(1,443)_ = 7.000, *p* = 0.008, partial η^2^ = 0.016] than AA homozygotes.

#### Objective Sleep Efficiency, Wake Bouts, and Wake After Sleep Onset Differed by CNR1 rs806368 Genotype at Baseline and Follow-Up in Participants With AUD

**Figure 3:**
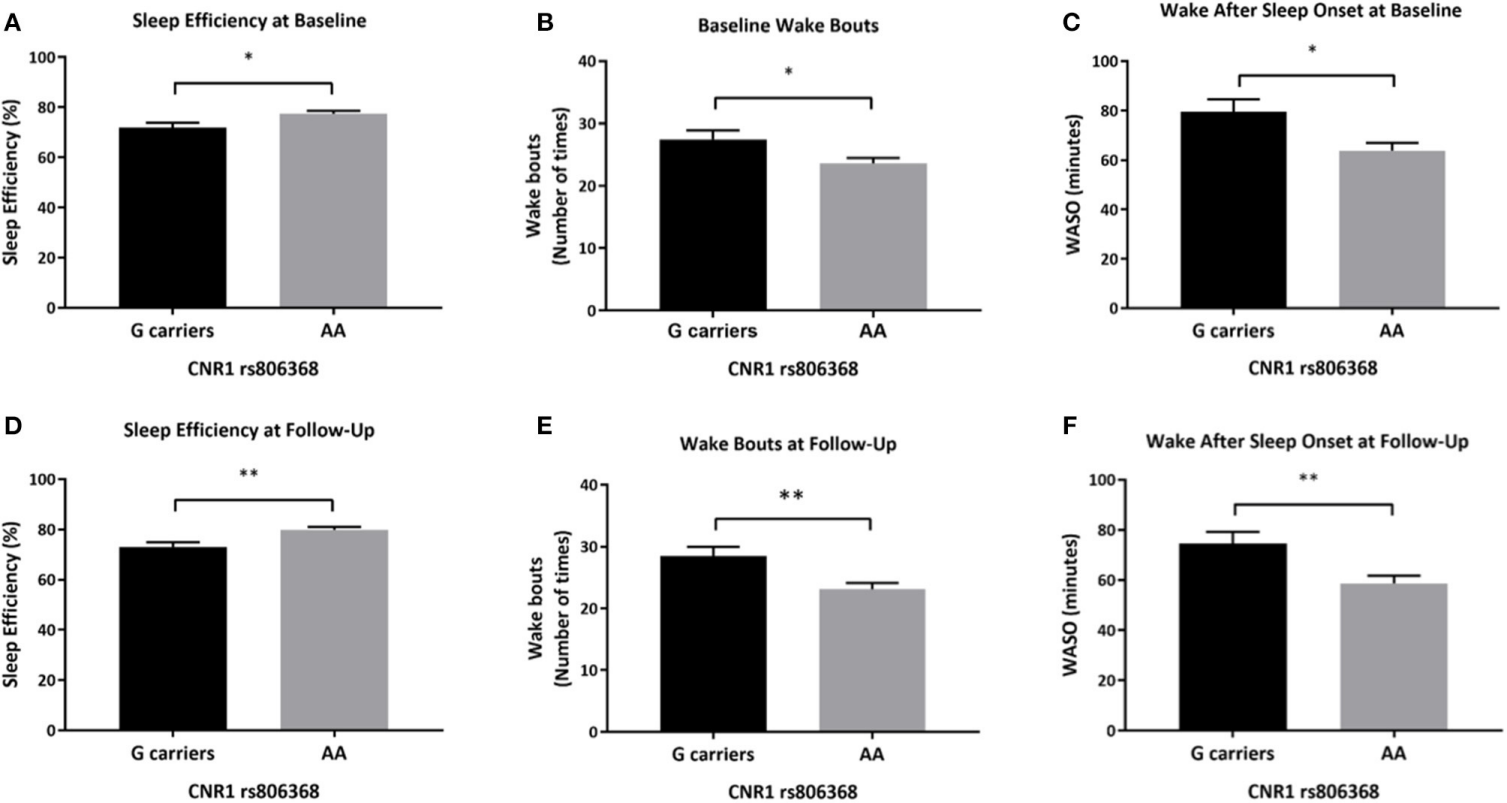
Differences in actigraphy-based sleep quality measures by CNR1 rs806368 genotype at baseline and follow-up. This figure demonstrates that at based on actigraphy-based measures, both at baseline **(A–C)** and follow-up **(D–F)**, G carriers of *CNR1* rs806368 genotype had poorer sleep efficiency, more wake bouts and more wake minutes after sleep onset after controlling for age, sex, years of education, pack years, Alcohol Dependence Severity (ADS) scores, Ancestry-informative marker (AIM scores for Africa, Europe), presence of mood disorders, and/or anxiety disorders, presence of Cannabis Use Disorder, and/or any Substance Use Disorder other than nicotine. **(A)** Baseline Sleep Efficiency: [*F*_(1,89)_ = 5.324, *p* = 0.023, *N* = 101], (**B**) Baseline wake bouts: [*F*_(1,89)_ = 4.952, *p* = 0.029, *N* = 101], **(C)** Baseline WASO: [*F*_(1,89)_ = 6.174, *p* = 0.015, *N* = 101], **(D)** Follow-up Sleep Efficiency *F*_(1, 65)_ = 9.052, *p* = 0.004, **(E)** Follow-up wake bouts: [*F*_(1, 65)_ = 8.125, *p* = 0.006], **(F)** Follow-up WASO: [*F*_(1, 65)_ = 7.677, *p* = 0.007]. Bars represent adjusted means and error bars denote standard errors. WASO, Wake After Sleep Onset. *p <0.05, **p <0.01 and Bonferroni adjusted.

After adjusting for covariates, there was a significant main effect of *CNR1* rs806368 genotype on actigraphy-derived measures including sleep efficiency, wake bouts and WASO (sleep efficiency: [*F*_(1,89)_ = 5.324, *p* = 0.023], partial η^2^ = 0.056, wake bouts: [*F*_(1,89)_ = 4.952, *p* = 0.029, partial η^2^ = 0.053], WASO: [*F*_(1,89)_ = 6.174, *p* = 0.015, partial η^2^ = 0.065]. Individuals carrying G allele of *CNR1* rs806368 had lower sleep efficiency [mean difference of −5.56% (95% CI, −10.35 to −0.77), *p* = 0.023], more wake bouts [mean difference of 3.9 bouts (95% CI, 0.4–7.3), *p* = 0.029] and more minutes awake after sleep onset [mean difference of 15.6 min (95% CI, 3.132 to 28.134), *p* = 0.015] (Figures 3A–C).

Similarly, at follow-up, there was a significant main effect of *CNR1* rs806368 genotype on sleep efficiency, wake bouts and WASO (sleep efficiency: [*F*_(1,65)_ = 9.052, *p* = 0.004, partial η^2^ = 0.122], wake bouts: [*F*_(1,65)_ = 8.125, *p* = 0.006, partial η^2^ = 0.111], WASO: [*F*_(1,65)_ = 7.677, *p* = 0.007, partial η^2^ = 0.106]. Individuals carrying the G allele of *CNR1* rs806368 genotype had lower sleep efficiency (mean difference of −6.19% (95% CI, −11.34 to −2.29), *p* = 0.004), more wake bouts [mean difference of 5.4 bouts (95% CI, 1.6–9.1), *p* = 0.006] and greater wake minutes after sleep onset [mean difference of 15.9 min (95% CI, 4.4–27.4), *p* = 0.007] (Figures 3D–F). Mean scores, unadjusted and adjusted for covariates are provided in Supplementary Table 3.

#### Objective Sleep Duration Differed by FAAH rs324420 Genotype at Follow-Up in Participants With AUD

**Figure 4:**
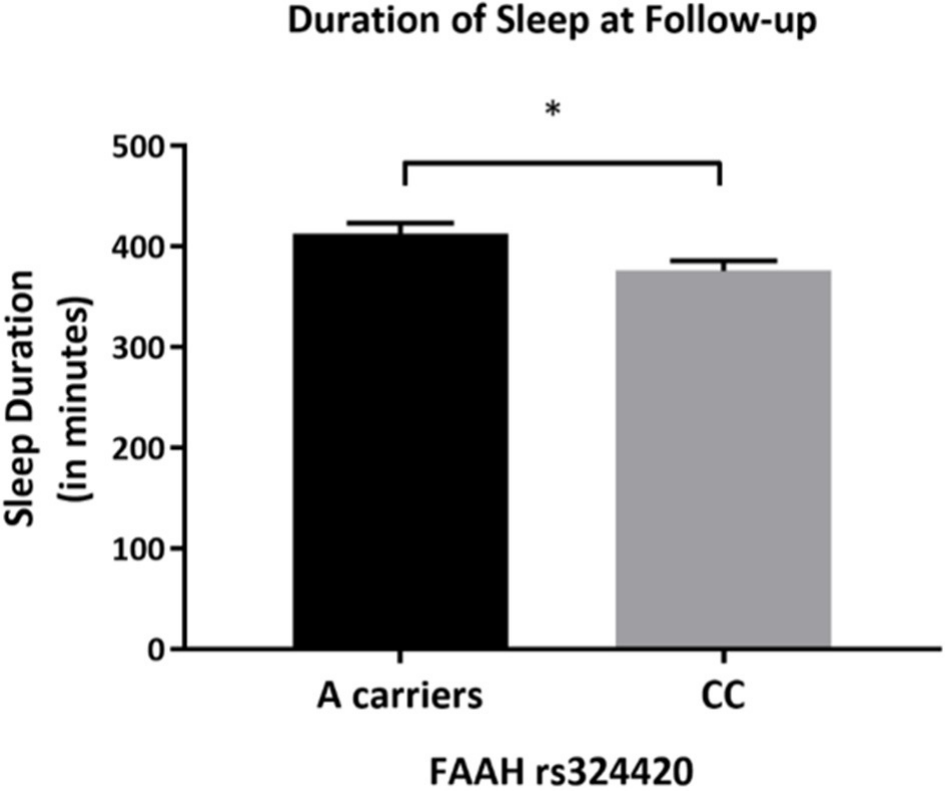
Differences in actigraphy-based sleep quality measures by FAAH rs324420 genotype at follow-up. This figure demonstrates that at follow-up, the CC *FAAH rs324420* genotype had lower sleep duration (based on actigraphy) after controlling for age, sex, years of education, pack years, Alcohol Dependence Severity (ADS) scores, Ancestry-informative marker (AIM scores for Africa, Europe), presence of mood disorders, and/or anxiety disorders, presence of Cannabis Use Disorder, and/or any Substance Use Disorder other than nicotine [*F*_(1, 65)_ = 5.944, *p* = 0.018]. Bars represent adjusted means and error bars denote standard errors. *p < 0.05.

After controlling for covariates, there was a statistically significant difference in actigraphy recorded sleep duration between the *FAAH rs324420* genotypes [*F*_(1,65)_ = 5.944, *p* = 0.018, partial η^2^ = 0.084]. Individuals homozygous for C allele had lower sleep duration (mean difference of 36.541 min (95% CI, −66.474 to −6.607), *p* = 0.018) than A carriers (Figure 4).

### If There Are Genetic Differences, Are They Mediated by Anxiety and Depressive Symptoms or Related Personality Traits?—Neuroticism Mediates the Relationship Between CNR1 rs6454674 Genotype and Sleep Disturbances Only in Controls

**Figure 5:**
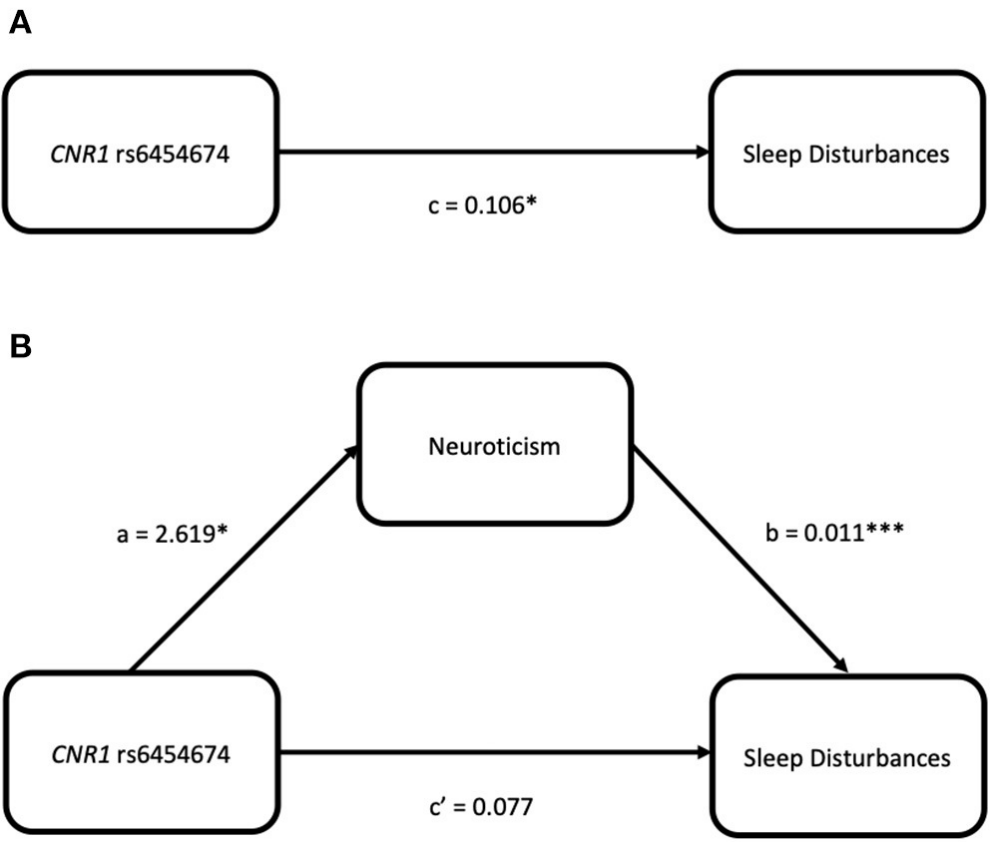
Simple mediation models for *CNR1* rs6454674 and sleep disturbances (from the PSQI) via neuroticism (from the NEO-PI-R). **(A)** Depicts the significant effect of genotype on sleep disturbances. **(B)** Indicates the significance is dropped when neuroticism is added as a mediator, suggesting neuroticism fully mediates the relation between *CNR1* rs6454674 genotype and sleep disturbances.

*CNR1* rs6454674 genotype influenced the sleep disturbances subscale of PSQI indirectly through its effects on the neuroticism domain of NEO-PI-R (ab = 0.028, bootstrap CI = 0.005–0.058). C carriers of *CNR1* rs6454674 genotype reported greater neuroticism scores (*a* = 2.619), with greater neuroticism scores associated with greater sleep disturbances (*b* = 0.011). There was no definitive evidence that *CNR1* rs6454674 genotype directly influenced sleep disturbances independent of this mediation (c' = 0.077, *p* = 0.125, 95% CI = −0.022 to 0.176) (Figures 5A,B). Model coefficients are provided in Table 3.

**Table 3:** Model coefficients for neuroticism mediation.

**Antecedent**		**Consequent**						
		***M*** **(Neuroticism)**				***Y*** **(Sleep disturbances)**		
		***Coeff*.**	***SE***	***p***		***Coeff*.**	***SE***	***p***
*X* (CNR1 rs6454674 genotype)	*a*	2.619	1.052	0.013	*c'*	0.077	0.050	0.125
*M* (Neuroticism)		–	–	–	*b*	0.011	0.003	<0.001
Constant	*i_*M*_*	43.557	0.780	<0.001	*i_*Y*_*	0.331	0.118	0.005
			*R*^2^ = 0.018				*R*^2^ = 0.061	
			*F*_(1,340)_ = 6.193, *p* = 0.013				*F*_(2,339)_ = 10.986, *p* < 0.001	

*X, Predictor; M, Mediator; Y, Outcome; Coeff., Coefficient; SE, Standard Error; a = path from X to M, c' = path from X to Y controlling for M, b = path from M to Y controlling for X*.

## Discussion

Our results highlight significant associations between endocannabinoid genotypes and subjective and objective sleep quality measures in individuals with AUD and controls. We performed a genetic association analysis with *CNR1* (rs806368, rs1049353, rs6454674, rs2180619) and *FAAH* (rs324420) SNPs and PSQI-based subjective and actigraphy-derived objective sleep measures in participants with AUD compared to non-AUD controls. This analysis identified associations between *CNR1*/*FAAH* genotypes and alcohol-associated sleep quality both in AUD and controls. C carriers of *CNR1* rs6454674 genotype were associated with one of the PSQI subscales (sleep disturbances) in both AUD and controls, and neuroticism mediated this relationship in controls. *CNR1* rs806368 and *FAAH* rs324420 genotypes were associated with objective sleep quality measures. We did not observe any differential distribution of genotypes between individuals with AUD and controls. Sleep disturbances associated with AUD do not always recover completely with abstinence (3), and are also may be a risk factor for relapse to drinking. Therefore, identifying possible genetic associations is vital to understand the underpinnings of alcohol-associated sleep disturbances.

We observed low-to-moderate correlations among global sleep quality and the alcohol consumption measure in both AUD and control groups. Whether these associations are alcohol exposure-related or the sleep disturbances preceded the alcohol use is difficult to ascertain from this study. However, we found correlations among global sleep quality and drinks per day, ADS score, and AUDIT total scores in the AUD group were stronger than in the control group. This suggests that the relationship between alcohol and poor sleep quality is more pronounced in AUD. As the AUD group drank more on average, these findings are consistent with the majority of previous studies describing the exposure-dependent effects of alcohol on sleep-related measures (67, 68).

Considering the subjective sleep measures and the genetic variations in the endocannabinoid pathway, we observed associations primarily with the PSQI subscale measure of sleep disturbance, wherein C carriers of the *CNR1* rs6454674 genotype reported greater disturbances than AA homozygotes. This finding was observed both in participants with AUD and in controls. While no study has previously reported differences in sleep quality associated with *CNR1* rs6454674 thus far, this polymorphism has previously been associated with various substance use disorders, including cocaine (69), cannabis (70), and also alcohol (59). In particular, the number of risk alleles (C) is associated with substance use disorder (SUD), especially in the European American population (71). Though not a study on sleep measures, Marcos et al. reported that the A allele associated with lower risk for sleep disturbances in our study has lower likelihood for AUD risk (59). Whether the risk for poor sleep predisposes the risk for AUD or vice versa cannot be deduced from this study. However, the findings from the control population suggest a possibility that the risk for sleep disturbances associated with this polymorphism could precede the development of AUD.

We had an interesting finding that the neuroticism personality domain mediates the association between *CNR1* rs6454674 polymorphism and sleep disturbances subscale in PSQI only in controls. Neuroticism domain is known to be associated with a tendency to experience negative affect and higher scores in this domain are known to be associated with many psychiatric disorders including substance use disorders (72). Juhasz et al. reported association of *CNR1* genotypes with depression, stating that the *CNR1* polymorphisms, specifically rs1049353, rs806366, could explain 1.5% of the variance in the neuroticism domain (73). This association with higher neuroticism scores in combination with the lower scores on the agreeableness domain was discussed to be a possible risk factor for SUD. In line with this finding, we found the neuroticism personality domain acted as a mediator for *CNR1* polymorphism and sleep disturbances, albeit for an SNP (rs6454674) that was different than the previously reported variants. Although we examined rs1049353 and rs806368 SNPs in our study, we did not find any association between these SNPs and sleep measures, hence we did not perform mediation analysis for these variants. Neuroticism is a known risk factor for AUD, and its association with sleep disturbances prompts the need for future research to explore whether personality domains act as contributing factors for sleep disturbances associated with alcohol. This could help to elucidate the complex etiology of sleep disturbance in AUD.

We observed consistent associations between *CNR1* genotypes and objective sleep measures both at baseline and follow-up among individuals with AUD. Among participants with AUD, *CNR1* rs806368 genotypes were associated with three actigraphy outcomes: sleep efficiency, number of wake bouts during sleep, and WASO minutes. Previously, *CNR1* rs806368 was reported to be associated with cannabis (74), cocaine (69), nicotine dependence (61), and in interaction with rs6454674 is known to increase the risk for drug and alcohol dependence (71). No prior associations with sleep phenotypes have been described with *CNR1* rs806368, although this variant has been reported to be associated with increased waist-hip ratio, triglycerides, body mass index, and obesity (75), which are known risk factors for poorer quality sleep. Previously an interaction between variant alleles of rs6454674 and rs806368 was associated with increased risk for AUD (59), both of which were independently associated with poorer sleep quality in our study. However, we did not observe any interaction effects of these SNPs on sleep measures. Despite the relatively small subset of our study sample, our association findings in the actigraphy data suggest that endocannabinoid genetic variants are associated with poorer sleep efficiency (*CNR1 rs806368 G allele and C allele of FAAH rs324420)* in individuals with AUD. This consistency in findings at baseline and follow-up (even with abstinence from alcohol) indicates two possible mechanisms: first, that there is an underlying risk for sleep disturbances which could be genetically driven, and second, that the long-standing effect of alcohol on sleep architecture is not resolved with short-term abstinence. Future studies including a longer abstinence period, are needed to explore these potential underlying mechanisms.

We observed a significant association between *FAAH* rs324420 genotype and sleep duration among individuals with AUD at the follow-up time point only. The variant A allele of this SNP is known to reduce *FAAH* expression and associated with AUD. There is only one human study describing an association of this polymorphism with sleep phenotypes in cannabis users, where the CC genotype was reported to have more sleep problems than the A carriers (24). In line with this evidence, our findings indicate that increased endocannabinoid activity (associated with the variant allele) is associated with better sleep quality. Although many studies report that A allele is associated with increased risk for AUD, this might be an independent association to AUD risk rather than a risk mediated by poorer sleep quality.

The findings of this study must be interpreted in the context of its strengths and limitations. We used both subjective and objective measures of sleep quality, although the objective (actigraphy) data was in a subset of individuals with AUD. This study is largely exploratory and considering the modest sample size for a genetic analysis, requires replication by future studies. As such, our study exemplifies the first steps in the recent Maps to Mechanisms to Medicine or the M2M2M approach that has been proposed as the way of the future in the application of human genetics to propel the understanding and treatment of common human diseases. This paradigm starts with examination of genetics with a focus on the discovery of disease-associated variants, including fine-mapping variants in close linkage disequilibrium and functional information of associated variants. This information is then translated to a better understanding of the etiology and progression of disease mechanisms, with the notion that the knowledge of causal mechanisms informed by genetic evidence will drive better therapeutics. In our study we have examined a gene set associated with the endocannabinoid system associated with sleep phenotypes in AUD. As a next step, studies could utilize larger sample sizes to analyze genetic variants overlapping sleep and alcohol-related phenotypes from GWAS catalog, such as *rs1421085 (FTO), rs13135092 (SLC39A8), rs13107325 (SLC39A8), rs75120545 (LRPPRC, AC019129.1)*, and *rs17601612 (DRD2)* for further exploration of genetic variants of endocannabinoid system and sleep phenotypes in AUD. In addition, future studies examining the molecular mechanisms underlying the role of the *CNR1* variants examined in this study are also warranted. Though our findings are preliminary and of small magnitude, this only reinforces the potential role of ECS in the crossroads of alcohol and sleep and should be weighed over their immediate translational perspective.

In conclusion, we report associations between *CNR1*/*FAAH* polymorphisms and subjective/objective sleep quality in alcohol-associated sleep disturbances. In controls, neuroticism mediated the relationship between *CNR1* polymorphism and sleep disturbances. These interesting findings broaden our understanding of underlying potential risk factors for poor sleep in AUD and expands the current knowledge of genetic associations of alcohol-related sleep quality.

## Data Availability Statement

The datasets used in this study are not publicly available due to ethical concerns regarding patient privacy and original patient consent. The raw data supporting the conclusions of this article will be made available by requests directly to the corresponding authors.

## Ethics Statement

The studies involving human participants were reviewed and approved by NIH Intramural Institutional Review Board. The patients/participants provided their written informed consent to participate in this study.

## Author Contributions

SS designed the analytical approach, conducted the data analysis, interpreted the findings, and drafted the manuscript. NK contributed to the manuscript development and analytical revisions. AB and MK acquired and processed the sleep data. MS was responsible for data curation and assisted with data analysis. DG was the PI of the clinical protocol and supported the clinical data acquisition. CH contributed to the genotype data. VR and GW were responsible for overall study concept and design. NK, VR, AB, MS, GW, and DG provided critical revisions. All authors critically reviewed content and approved the final version for publication.

## Funding

This project was funded with federal funds from the National Institutes of Health, Clinical Center Intramural Research Program, and the National Institute on Alcohol Abuse and Alcoholism Division of Intramural Clinical and Biological Research (Z1A AA 000466). The data that support the findings of this study are available from the corresponding authors upon reasonable request.

## Conflict of Interest

The authors declare that the research was conducted in the absence of any commercial or financial relationships that could be construed as a potential conflict of interest.

## Publisher's Note

All claims expressed in this article are solely those of the authors and do not necessarily represent those of their affiliated organizations, or those of the publisher, the editors and the reviewers. Any product that may be evaluated in this article, or claim that may be made by its manufacturer, is not guaranteed or endorsed by the publisher.
