# Supplementary material for: FAAH and CNR1 Polymorphisms in the Endocannabinoid System and Alcohol-Related Sleep Quality
Source: Front Psychiatry. 2021 Sep 9;12:712178. doi: 10.3389/fpsyt.2021.712178 (PMC8458733; doi:10.3389/fpsyt.2021.712178)
Supplement: Supplementary file 1 [file Data_Sheet_1.docx]

**Supplementary Figure 1**

*Schema of the Study*

**
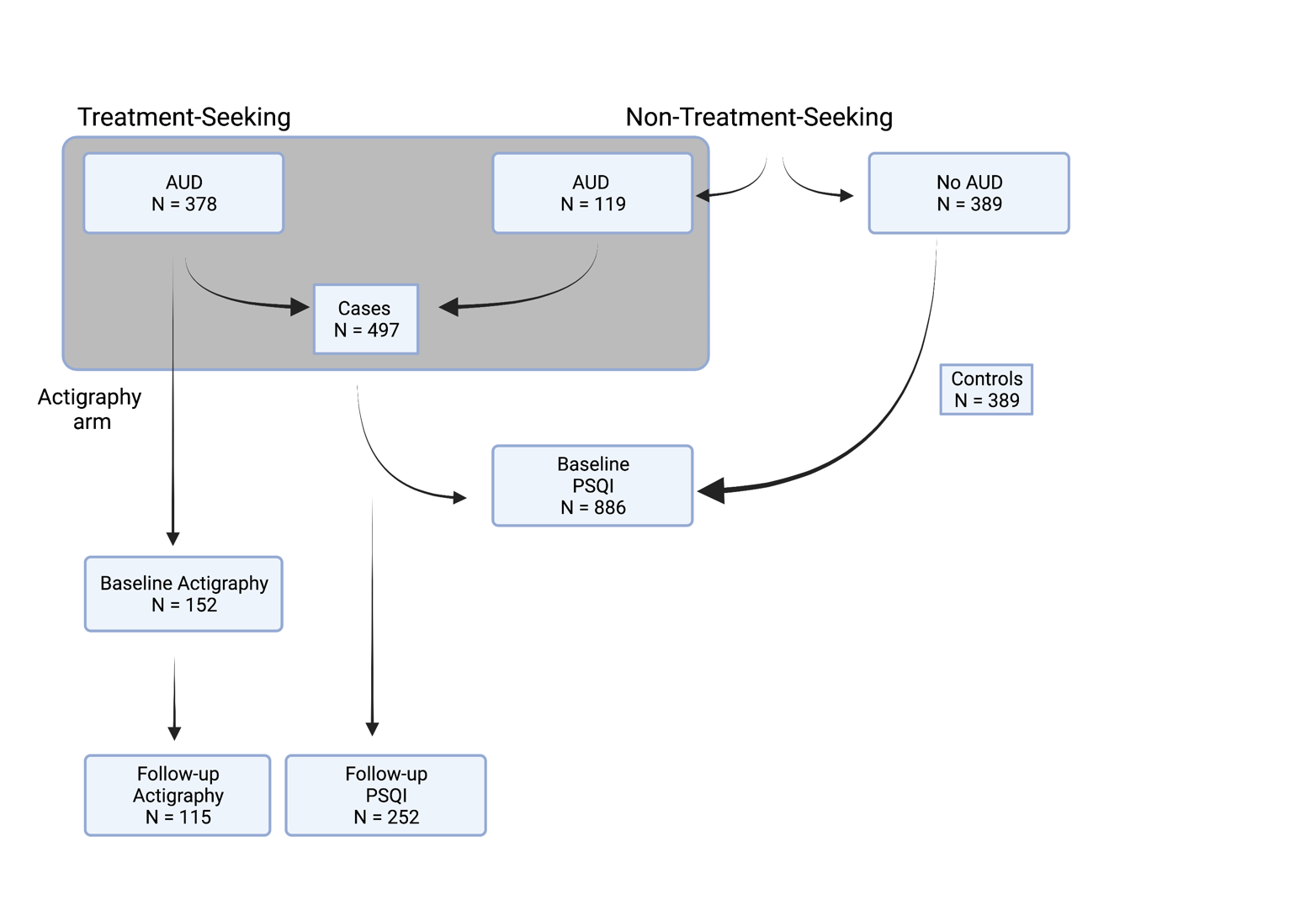
**

*Note.* This figure depicts the flow of the study. N denotes sample size. AUD (cases) participants from both treatment-seeking and non-treatment seeking groups are represented by a grey box. Baseline PSQI was available for both AUD and control groups, whereas follow-up PSQI was available only for 50% of AUD cases. An actigraphy arm included only 40% of the treatment-seeking group on week 1 for baseline actigraphy measures. A week-4 follow-up actigraphy measures were available for around 75% of the group that complete the baseline actigraphy assessment on week 1. Flowchart created in [BioRender.com](https://biorender.com/)

**Supplementary Figure 2**

*Research Questions and Statistical Approaches*

**
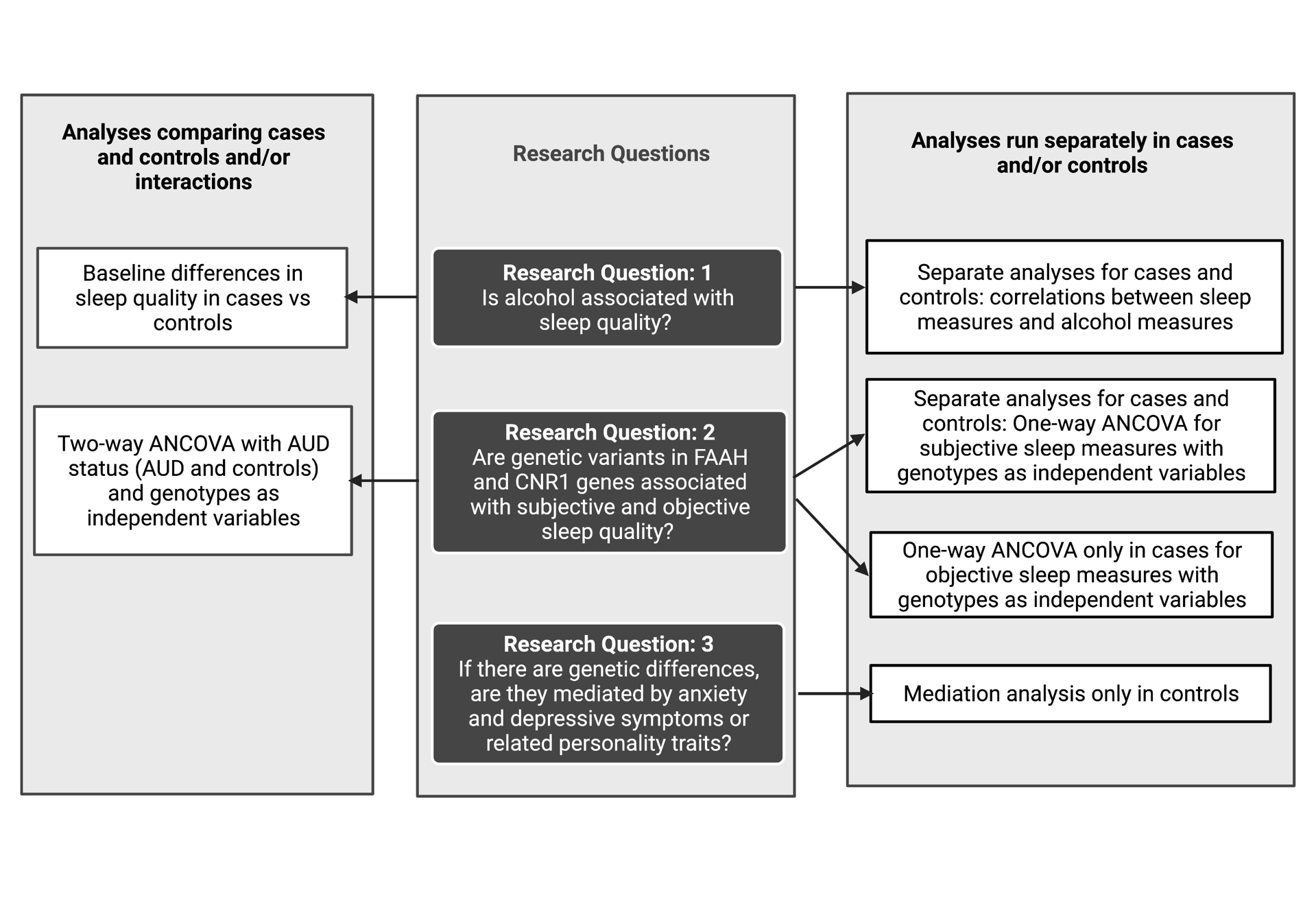
**

*Note.* This flowchart depicts the statistical approaches for all the research questions. Analyses comparing AUD cases and healthy controls (case vs control approach) and analyses including case status (AUD/no AUD) as one of the independent variables are grouped on the left. Analyses run separately for cases and controls, or only in cases (actigraphy data available only in cases) and only in controls (mediation analysis) are denoted on the right. Flowchart created in [BioRender.com](https://biorender.com/)

**Supplementary Figure 3**

*Two-way Interaction Between Alcohol Dependence Status and CNR1 rs1049353 Genotype*


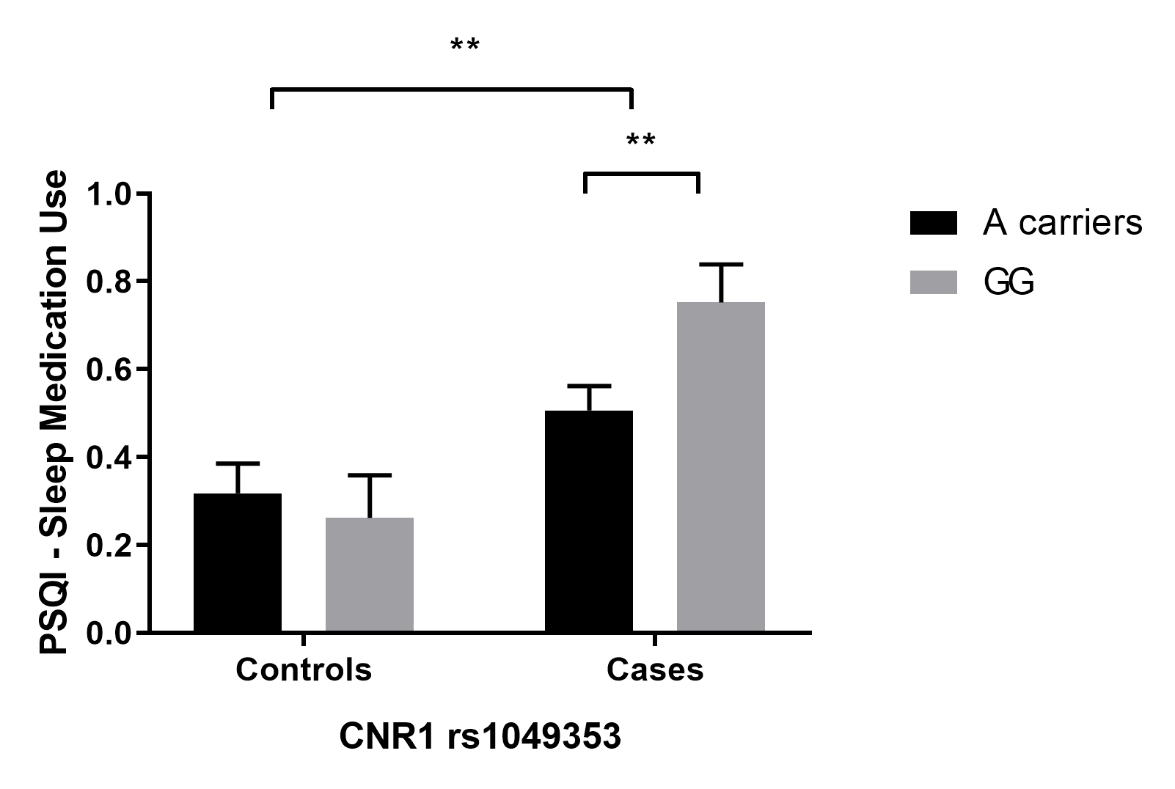


*Note.* This figure demonstrates the significant interaction between *CNR1* rs1049353 genotype and alcohol dependence status on sleep medication use after controlling for age, sex, years of education, pack years, ADS scores, AIM scores (Africa, Europe), mood disorders, anxiety disorders, cannabis use disorder, and any other substance use disorder other than nicotine (F (1,828) = 5.253, *p* = 0.022). There was a significant difference in sleep medication use between cases and controls (*p* = 0.001). Among cases, GG genotype reported more medication use than A carriers (*p*=0.008). Bars represent adjusted means and error bars denote standard errors.

PSQI = Pittsburgh Sleep Quality Index

** *p<0.01* and Bonferroni adjusted

**Supplementary Table 1**

*Comparison of Pittsburgh Sleep Quality Index (PSQI) scores among participants with AUD and controls at baseline and follow-up scores of only participants with AUD*

| **Components** | **Controls** | **AUD** | ***p* value ^a^** | **AUD ^b^** | ***p* value ^c^** |  |
| --- | --- | --- | --- | --- | --- | --- |
| **Baseline measures** | | | | **Follow-up** |  |  |
| **N** | **389** | **497** |  | **252** |  |  |
| 1. Sleep Duration 2. Sleep Disturbance 3. Sleep latency 4. Daytime dysfunction 5. Sleep efficiency 6. Overall sleep quality 7. Sleep medication use | 0.42±0.7  0.86±0.5  0.42±0.6  0.27±0.5  0.41±0.8  0.55±0.6  0.09±0.4 | 1.46±1.2  1.67±0.7  1.23±0.8  1.18±0.8  0.98±1.1  1.56±0.9  0.73±1.1 | <0.001  <0.001  <0.001  <0.001  <0.001  <0.001  <0.001 | 1.16±1.1  1.48±0.6  1.06±0.7  0.77±0.7  0.75±1.0  1.13±0.8  0.56±1.1 | <0.001  <0.001  <0.001  <0.001  <0.001  <0.001  <0.001 |  |
| Global score | 3.02±2.4 | 8.80±4.3 | <0.001 | 6.90±3.7 | <0.001 |  |

*Note.* Component scores range between 0-3, score “0” indicates no sleep difficulty and score “3” indicates severe sleep difficulty. Global score ranges between 0-21 with “0” indicating no difficulty and “21” severe difficulty. A global score of >4 indicates poor sleep quality

AUD = Alcohol Use Disorder

^a^ Controls vs AUD at baseline

^b^ Only participants with AUD receiving in-patient treatment were followed up (mean follow-up 26.09±1.9 days)

^c^ AUD baseline vs Follow-up

**Supplementary Table 2**

*Adjusted and unadjusted mean sleep disturbances among CNR1 rs6454674 genotype groups with covariates*

|  | Controls | | | | | AUD | | | | |
| --- | --- | --- | --- | --- | --- | --- | --- | --- | --- | --- |
| *CNR1* rs6454674 | *N* | *Unadjusted* | | *Adjusted* | | *N* | *Unadjusted* | | *Adjusted* | |
|  |  | *M* | *SD* | *M* | *SE* |  | *M* | *SD* | *M* | *SE* |
| AA homozygotes  C carriers | 158  199 | 0.790  0.910 | 0.480  0.463 | 0.791  0.910 | 0.037  0.033 | 188  268 | 1.60  1.74 | 0.690  0.692 | 1.599  1.740 | 0.044  0.037 |

*Note.* AUD = Alcohol Use Disorder; N = Number of subjects; *M* = Mean; *SD* = Standard Deviation; *SE* = Standard Error.

Covariates in the adjusted model: Age, sex, years of education, pack years, Alcohol Dependence Severity (ADS) scores, Ancestry Informative Markers for Africa & Europe, presence of mood disorders, presence of anxiety disorders, presence of cannabis use disorder, and any other substance use disorder other than nicotine

**Supplementary Table 3**

*Adjusted and unadjusted mean objective sleep quality measures among CNR1* rs806368 *genotype groups with covariates*

| Baseline | Sleep Efficiency (%) | | | | | Wake bouts (times) | | WASO (minutes) | | | | | | |
| --- | --- | --- | --- | --- | --- | --- | --- | --- | --- | --- | --- | --- | --- | --- |
| *CNR1* rs806368 | *N* | *Unadjusted* | | *Adjusted* | | *Unadjusted* | | | *Adjusted* | | *Unadjusted* | | *Adjusted* | |
|  |  | *M* | *SD* | *M* | *SE* | *M* | *SD* | | *M* | *SE* | *M* | *SD* | *M* | *SE* |
| AA homozygotes  G carriers | 72  29 | 77.29  72.32 | 8.6  12.8 | 77.47  71.91 | 1.19  1.97 | 24.02  26.49 | 7.35  5.97 | | 23.62  27.48 | 0.86  1.42 | 64.87  76.96 | 21.63  31.48 | 63.85  79.48 | 3.11  5.15 |
| Follow-up |  |  |  |  |  |  |  | |  |  |  |  |  |  |
| AA homozygotes  G carriers | 52  25 | 79.32  74.15 | 7.53  10.46 | 79.85  73.04 | 1.20  1.80 | 23.51  27.63 | 6.71  7.69 | | 23.10  28.47 | 0.99  1.49 | 59.93  72.18 | 19.72  25.85 | 58.74  74.65 | 3.05  4.57 |

*Note.* N = Number of subjects, *M* = Mean, *SD* = Standard Deviation, *SE* = Standard Error.

Covariates in the adjusted model: Age, sex, years of education, pack years, Alcohol Dependence Severity (ADS) scores, Ancestry Informative Markers for Africa & Europe, presence of mood disorders, presence of anxiety disorders, presence of cannabis use disorder, and any other substance use disorder other than nicotine
